# Behavioural Difficulties That Co‐occur With Specific Word Reading Difficulties: A UK Population‐ Based Cohort Study

**DOI:** 10.1002/dys.1496

**Published:** 2015-02-18

**Authors:** Ginny Russell, Denise Ryder, Brahm Norwich, Tamsin Ford

**Affiliations:** ^1^ Institute of Health Services Research, Medical SchoolUniversity of ExeterUK; ^2^ Graduate School of EducationUniversity of ExeterUK

**Keywords:** attention deficit hyperactivity disorder, comorbidity, developmental disorders, diagnosis, dyslexia

## Abstract

This study aimed to examine the association between specific word reading difficulties (SWRD) identified at age 7 years using a discrepancy approach and subsequent dimensional measures of behavioural difficulties reported by teachers and parents at age 11 years. Behavioural problems were assessed using the Strengths and Difficulties Questionnaire. Secondary analysis of a UK representative population‐based sample of children (*n* = 12 631) was conducted using linear regression models.

There were 284 children (2.2%) identified with SWRD at age 7 years. Children with SWRD had significantly higher scores on all measures of behavioural difficulties in unadjusted analysis. SWRD was associated with elevated behavioural difficulties at age 11 years according to parent report, and with greater emotional problems, hyperactivity and conduct issues according to teachers, even after having controlled for baseline difficulties. These results were replicated for children with low reading attainment, but no cognitive ability discrepancy. Categories of special educational need into which children with SWRD were classed at school were varied.

Given high rates of co‐occurring behavioural difficulties, assessment that identifies each individual child's specific functional, rather than categorical, difficulties is likely to be the most effective way of providing classroom support. © 2015 The Authors. *Dyslexia* published by John Wiley & Sons Ltd.

## Introduction

It has long been recognized that reading difficulties often co‐occur with a range of other developmental disorders (Fawcett, 2012). A frequent limitation of research is that it has focussed on groups of referred children, and is therefore subject to referral bias (Andrews, Wisniewski, & Mulick, 1997). Most studies have examined whether children's reading difficulties co‐occur with other diagnoses or classes of developmental disorder, thus behavioural problems without diagnosis are often overlooked. In this study, we avoid such problems by analysis of a population‐based sample, to assess whether children with specific word reading difficulties suffer from a range of co‐occurring behavioural problems. As children with reading difficulties are likely to be seen by education specialists, awareness and understanding of potential co‐occurring behavioural difficulties can be a great help in designing and managing interventions in the classroom and beyond (Norwich, 2014).

The Diagnostic and Statistical Manual of Mental Disorders (American Psychiatric Association & DSM‐5 Task Force, 2013), retains the notion of specificity in its definition of the general diagnostic category ‘specific learning disorder’. Strong emphasis is placed on academic performance discrepant with an individual's intelligence and age, as it is in the definition adopted by the *International Classification of Diseases* (ICD‐10, World Health Organization, 1993) in which ‘specific reading disorder’ is characterized by significant impairment in the development of reading skills, which is not solely accounted for by mental age, visual acuity problems or inadequate schooling. Despite ongoing controversy around this discrepancy concept (Elliott & Grigorenko, 2014), many UK researchers continue to adopt a discrepancy definition in research studies. It was a version of this discrepancy definition that Rutter and Yule (1975) used in their influential UK study of specific reading retardation amongst children in the Isle of Wight. These authors also applied a cutoff point of 2 standard deviations below prediction of reading ability to large populations of 10 year old readers in two different socioeconomic regions of the UK. They found that 3.9% (mixed rural area) and 9.9% (deprived inner city area) of children fell into the category of specific reading retardation (Berger, Yule, & Rutter, 1975), little different from current UK prevalence estimates of between 3% and 10% cited by Snowling and Hulme (2012).

More recently, Ferrer, Shaywitz, Holahan, Marchione, and Shaywitz (2010) produced empirical evidence for what they describe as an uncoupling between cognition and reading in dyslexic, as opposed to non‐dyslexic, readers. The two abilities are not always interlinked in dyslexic readers: this, they claim, is an evidence of what has historically been the core concept of dyslexia, which remains the focus of most current defining models of specific reading difficulties. Not surprisingly, then, in terms of research practice, the use of IQ discrepancy‐defined groups has been, and remains, the usual approach when studying specific word reading difficulty (Hulme & Snowling, 2009), as distinct from non‐discrepant low reading attainment. This is despite formal UK educational policy guidelines, which state that ‘dyslexia’ occurs across the range of intellectual abilities, is best thought of as a continuum, and has no clear cutoff points (Rose, 2009). The discrepancy model's gradual decline in education, as opposed to research, has come about largely because all poor readers have been shown to benefit from similar intervention, at least in the early stages (Vellutino, Scanlon, Small, & Fanuele, 2006).

#### Co‐occurring behavioural problems

Many studies have examined concurrent behavioural issues that may affect children with reading difficulty. Most of these have adopted medical terminology with the use of other diagnostic categories of disorder (Gillberg, 2010). Cheung *et al.* (2012) found reading difficulties co‐occurred with attention deficit hyperactivity disorder (ADHD), with over half (53–72%) of the overlapping familial influences between ADHD and reading difficulties not shared with IQ. In a Scandinavian school based study, Kadesjö and Gillberg found 40% of children with ADHD showed reading problems and 29% writing problems (2001), albeit using a very small sample (*n* = 15). Cheung *et al.* (2012) review three studies in the field that estimate 25–40% of individuals with ADHD having reading disorder or vice versa. Further evidence of co‐occurrence comes from studies of shared ADHD symptoms (inattentiveness and hyperactivity) and reading ability/disability as continuous traits in population samples (Paloyelis, Rijsdijk, Wood, Asherson, & Kuntsi, 2010). Reading difficulties frequently co‐occur with a range of other developmental disorders, including developmental coordination disorder (Lingam *et al.,* 2010) and conduct disorder (Hinshaw, 1992). Kaplan, Dewey, Crawford, and Wilson (2001) studied a population‐based sample of 179 children receiving special support in Calgary. If the children met dyslexia criteria, there was a 51.6% chance of having another disorder. If the children met the ADHD criteria there was an 80.4% chance of having another disorder.

Several studies have shown reading failure co‐occurs with aberrant behaviour other than hyperactivity. In two UK population‐based samples emerging from the Isle of Wight and an Inner London Borough (Berger *et al.,* 1975), there was a strong tendency for the children with specific reading difficulties to have a high rate of other behavioural problems at school. Some UK studies have examined the extent to which behavioural difficulties coexist with reading disability, but most, like the Isle of Wight study, were conducted some time ago (e.g. McGee, Williams, Share, Anderson, & Silva, 1986). Two more recent US studies have emphasized the phenomena of co‐occurrence. Morgan, Farkas, Tufis, and Sperling (2008) found that US children with reading problems at age 7 years were more likely to display poor task engagement, poor self‐control, externalizing and internalizing behaviour problems 2 years later. More recently, Dahle and colleagues (2010) examined behavioural problems in children with severe dyslexia. They found more behavioural problems in the group with severe dyslexia than in controls, in all areas measured. In addition, parents reported more children with dyslexia to be anxious and depressed and have social problems and attention problems than teachers did.

Frith and Happé (1998) have suggested that co‐occurrence of developmental disorders may be due to downstream effects–where one difficulty exacerbates another (e.g. a tendency to reading difficulty is amplified by inattention). Other theorists have suggested one cognitive deficit (such as slow processing speed) may underlie several symptomatic behaviours in a range of developmental disorders (Bental & Tirosh, 2007). It is plausible that the same underlying genetic or neurological mechanisms may underlie co‐occurrence of dyslexia and other developmental disorders such as ADHD and autism spectrum disorder (ASD) as suggested by Reiersen, Constantino, Grimmer, Martin, & Todd (2008).

We assessed co‐occurrence of behavioural problems in a group of children with specific word reading difficulties (SWRD) using a population‐based sample

Our objectives were as follows:

1. To establish whether later behavioural problems at age 11 years were elevated in the group with SWRD as opposed to typical reading ability, after accounting for initial behavioural differences at age 7 years.
2. To examine the proportion of children with a diagnosis ASD and/or ADHD.
3. To report categories of Special Educational Needs (SEN) for children with SWRD and analyse how recognition of SEN related to extent of behavioural and reading difficulties.

Because of the controversy over use of the discrepancy definition (*e.g.* Elliott & Grigorenko, 2014), we also demarcated a group who had low word reading attainment (LWRA) based on age rather than cognitive ability. Additional sensitivity analysis was repeated on children with and without LWRA.

Despite the critique of the discrepancy definition described previously, we were interested in specific word reading difficulty, ‘unexpected’ underachievement in word reading skills. There have been numerous studies indicating a link between childhood behaviour problems, mental disorder and intellectual disability (see Einfeld, Ellis, & Emerson, 2011, for systematic review). We aimed to test whether word reading difficulties in children with typical IQ might also be associated with behavioural problems. Therefore, we chose to examine the association between wider behavioural difficulties and specific reading problems that were not synonymous with intellectual disability. Also, in using the discrepancy criteria, direct comparison was possible with a similarly large, albeit much earlier, epidemiological study of British children's reading difficulties (Rutter & Yule, 1975).

Because of the persistent questions over use of the discrepancy definition, we also backed up our findings with sensitivity analyses using a group defined without discrepancy. We expected differences in problematic behaviour to be amplified in this group as research has shown children with lower cognitive ability often have co‐occurring behavioural problems.

## Methods

### Sample

The Millennium Cohort Study (MCS) is the fourth of the UK's national longitudinal birth cohort studies (University of London, 2008). Each follows a large sample of individuals, born over a limited period of time, through the course of their lives, charting the effects of events and circumstances in early life on outcomes and achievements later on. The cohort is a UK‐representative birth cohort study using a disproportionate stratified cluster sampling design. Children born between 1 September 2000 and 11 January 2002 and listed on the Child Benefit Records were eligible for the study. Child Benefit is a financial benefit payable to all parents of UK children, with near universal take up. Sampling of electoral wards (the clusters) were stratified by UK country (England, Scotland, Wales and Northern Ireland), and further stratified by ethnic group composition (whether at least 30% of the population fell into the categories ‘Black’ or ‘Asian’) and level of child poverty in England, and by level of social disadvantage in Scotland, Wales and Northern Ireland (Hansen & Joshi, 2010). Data were first collected when children were 9 months old (1st sweep, 18 519 participating families), and further data were recorded concerning the children's health and development when the children were 3 years old (2nd sweep, *n* = 15 590), 5 years old (3rd sweep, *n* = 15 246), 7 years old (4th sweep, *n* = 13 857) and 11 years old (5th sweep, *n* = 12 026). Details of sampling design are documented in detail elsewhere (Plewis, 2007).

The attrition and selection analyses were done on MCS data ‘in house’ by the MCS curators, and details are openly available to researchers. MCS curators provide standardized weightings that account for selective attrition in the sample over time. The way in which weightings were derived at each sweep is described in MCS documentation (Plewis, 2007). The MCS standardized weightings were used in analysis to account for the effects of attrition at sweeps 4 and 5 and to adjust results to be representative of the UK population as a whole. Consistent with other studies using these data (Totsika, Hastings, Emerson, Berridge, & Lancaster, 2011), families with twins or triplets were excluded (252 twins and 11 triplets) as the outcome would be expected to be correlated within families. We also excluded children for whom English was not the first language. MCS interviewers did not to run the tests if the child had a very serious learning disability/behavioural problem or was unable to respond to the assessment by pointing or speaking. This was likely to have resulted in the exclusion of a very small number of children with severe intellectual disability.

Ethical protocols for MCS including policy on informed consent and anonymity for MCS participants are detailed in Shepherd (2012). Ethical approval for the current study was granted by the School of Social Science Ethics Committee at the University of Exeter. MCS data are freely available to accredited researchers.

### Measures

Word reading ability was measured in face‐to‐face assessments for each child (*n* = 13 423) using the second edition of the British Ability Scales (BAS) battery (Elliott, Smith, & McCullock, 1996). The child read aloud a series of words presented on a card. The assessment consists of 90 words in total. The words are organized into 9 blocks of 10 words in ascending order of difficulty. The child is asked to read out loud each word in a block to the interviewer. The number of blocks of words the child is asked to attempt to read is dependent on the child's performance during the assessment. This assessment is designed to be used with children aged from 5 years to 17 years and 11 months. All of the children assessed in MCS sweep 4 started at the first item, as this was the starting point for children of their age. A child's progression through the assessment is dependent on the number of words they read correctly. If a child makes 8 errors in a block of 10 words, then the assessment stops. Word reading scores were adjusted for age in three month blocks. Internal reliability of the BAS Word Reading Scale is excellent (Cronbach's alpha 0.93).

#### The specific word reading difficulties group

Table 1 provides a timeline showing at what ages various measures used in the study were taken. In this study, our primary analysis adopted a discrepancy definition of specific word reading difficulties, for pragmatic identification of research subjects, despite its known shortcomings (see Hulme & Snowling, 2009, p.37–39 for discussion). We defined specific word reading difficulties by looking at the difference between word reading scores predicted according to cognitive ability and actual observed word reading scores. General Cognitive Ability (GCA) was derived from core scores of the BAS scales. *T*‐scores for verbal ability (BAS naming vocabulary), visual ability (BAS pattern construction) and reasoning (BAS picture similarities score) were averaged to compute BAS GCA scores.

**Table 1:** Timeline of when measures were taken in Millennium Cohort

	Sweep 3	Source	Sweep 4	Source	Sweep 5	Source
Child mean age (years)	5.2 years		7.2 years		11 years	
Measures taken	BAS naming vocabulary tests	Cognitive test	BAS word reading tests	Cognitive testing		
	BAS pattern construction tests	Cognitive test	Strengths and Difficulties Questionnaire	Teacher and parent rated	Strengths and Difficulties Questionnaire	Teacher and parent rated
	BAS picture similarities tests	Cognitive test	ASD and ADHD diagnosis	Parent report		
			School report of SEN category	Teacher report		

BAS, British Ability Scales; ASD, autism spectrum disorde; ADHD, attention deficit hyperactivity disorder; SEN, Special Educational Needs.

Description of measure that make up GCA

- The BAS naming vocabulary test assesses the spoken vocabulary of young children. The test items consist of a booklet of coloured pictures of objects, which the child is shown one at a time and asked to name. The scale measures expressive language ability, and successful performance depends on the child's previous development of vocabulary of nouns. Internal reliability is good (Cronbach's alpha 0.75).
- The BAS pattern construction assessment tests spatial awareness. The child constructs a design by putting together flat squares or solid cubes with black and yellow patterns on each side. The child's score is based on accuracy and speed. Internal reliability is very good (Cronbach's alpha 0.83).
- The BAS picture similarities test measures children's problem solving abilities. Children are shown a row of four pictures on a page and asked to place a card with a fifth picture under the picture most similar to it. Internal reliability is very good (Cronbach's alpha 0.81).

The BAS manual (Elliott *et al.,* 1996; Table 5.3, pp. 438–439) gives a table for conversion of summed and mean *t*‐scores to GCA. The BAS scores were recorded at age 5 years (mean age 5.2 years, standard deviation [SD] = 0.23, range 4.4–6.1 years) for all children in MCS using face‐to‐face BAS tests administered in each child's own home by trained interviewers. Word reading ability was measured later (at mean age 7.2 years, SD = 0.25, range 6.3–8.4 years), again using face‐to‐face BAS tests administered in children's own homes and converted to standard age adjusted scores according to BAS guidelines.

**Figure 1:**
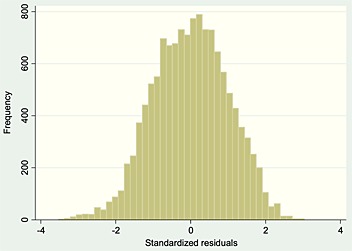
Plot of standardized residuals showing deviation in word reading scores from that predicted by General Cognitive Ability (*n* = 12 630).

We defined specific word reading difficulties using linear regression with word reading (ability) score as the outcome and our proxy for IQ (GCA) as the predictor. Plotting the residuals from this regression gives a distribution showing where the actual word reading score deviates substantially from that predicted by GCA (Figure 1). We followed the method of Rutter and Yule (1975) that used a cutoff of minus 2 SDs on these residual scores to define a group with severe word reading disability. They argued that this method is the only satisfactory means of taking into account regression to the mean effect when assessing under (or over) achievement. Furthermore, the BAS technical manual provided analyses (Table B9, BAS manual) using the same regression approach, and this is the method of defining discrepancy recommended by the authors. Individuals in the SWRD group had substantially lower reading scores than were predicted by their CGA.

#### The low word reading attainment group

Children with LWRA were defined without reference to GCA discrepancy. Children included were those with word reading attainment less than 2 SD below the mean on age adjusted scores at age 7 years.

#### Measure of behavioural problems

The Strengths and Difficulties Questionnaire (SDQ) (Goodman, 2001) is a brief dimensional measure of behavioural problems amongst children aged 4–16 years, which has been widely adopted in both research and practice (Russell, Rodgers, & Ford, 2013). The instrument is composed of 25 items that ask about behavioural attributes of the child, and these are combined to form five subscales (composed of 5 items each). The subscales measure emotional symptoms, conduct problems, hyperactivity/ inattention, peer relationships and prosocial behaviour. There are parallel versions of the SDQ that collect the same data from parents, teachers and young people aged 11 years or over. A supplemental ‘impact’ subscale measures chronicity, distress, social impairment and burden to others, which provides useful additional information for clinicians and researchers (Goodman, 1999).

The SDQ impact scales measures the frequency of distress, consequent social impairment, whether the problems interfere with classroom learning, family life, and peer relationships and the burden to the teacher/parent and the child themselves and the class as a whole. SDQ subscales result in generation of scores between 1 and 10 with higher scores symbolizing greater difficulties. Prosocial scores are the exception to this as increasing score represented decreasing impairment. Details of the questionnaire items that make up the SDQ, normative data, background research and how the subscales are scored are available at the SDQ website (www.sdqinfo.org). In MCS, each SDQ subscale was recorded for the entire cohort at sweep 4 (mean age 7 years) and sweep 5 (mean age 11 years) from both parent and teacher informants, except the ‘impact’ subscale, which was only recorded at sweep 4. The internal reliability of the SDQ measures have been assessed in several studies (*e.g.* Mieloo *et al.,* 2012). The internal reliability of the SDQ scores ranged between 0.5 and 0.8 in all the SDQ subscales used in this study. The hyperactivity and prosocial subscales had the greatest internal reliability. This indicates that the items on each SDQ subscale are measuring the same underlying latent construct.

#### Parent‐report of autism spectrum disorder/attention deficit hyperactivity disorder diagnosis

At sweep 4, parents or carers were asked if a doctor or health professional had told the parent that their child had ADHD or ASD. Families at sweep 4 who responded with positive or negative answers to the question were included. Families who answered ‘don't know’ or refused to answer were excluded from the relevant analysis. Forty‐four children had a dual diagnosis of ASD and ADHD. In total, from this sample, 180 children had reportedly been identified with ADHD and 209 with ASD (Russell, Rodgers, Ukoumunne, & Ford, 2013).

#### School report of Special Educational Needs

The teacher survey took the form of a postal self‐completion questionnaire sent to a named teacher at the child's school. The head teacher of the school also received an information pack containing a cover letter and a survey leaflet at the same time as teachers were first sent the questionnaire. Information was returned by teachers for 70% of children at sweep 4 (*n* = 8876; at age 7 years). As part of this survey, teachers were asked if the child had ever been recognized as having SEN. For those with SEN, they were also asked whether the child had problems in any of the following areas:

- Dyslexia
- Learning difficulties (including dyspraxia/dyscalculia)
- Attention deficit and hyperactivity disorder (ADHD)
- Autism, Asperger's syndrome or autistic spectrum disorder
- Other difficulties with reading, writing, spelling or maths
- Problem with speech or language
- Problem with sight
- Problem with hearing
- Mental illness/depression

### Analysis

Mean scores for children's behaviours at ages 7 and 11 years were compared on the SDQ subscale scores in the SWRD groups and the sample without SWRD. Linear regression *t*‐tests were used to compare differences between the groups. This model used SDQ subscales as the dependent variable and SWRD as a grouping variable, so it examined to what extent children with and without specific word reading difficulties differed on all the behavioural subscales as reported both by parents and teachers. Linear regression is equivalent to analysis of variance for dependent continuous variables, as in this case (SDQ behaviour scales were our outcomes and these were treated as continuous), as it compare means/variance of outcomes by groups (in this case SWRD or typical reading ability).

The analysis was repeated again with SDQ behaviour scores at age 11 years as the dependent variable and SWRD as the independent variable, but adjusting for initial behavioural difficulties at age 7 years (the covariate). This was to determine whether children with SWRD developed more behavioural problems later in childhood even after the initial level of behavioural difficulty at age 7 years was controlled for.

Both adjusted and unadjusted models were run several times, once for each subscale. For example, we looked for differences between children with and without SWRD in parent‐reported hyperactivity at age 11 years having controlled for parent‐reported hyperactivity at age 7 years: a separate analysis tested differences between children with and without SWRD in teacher‐reported emotional symptoms at age 11 years having accounted for teacher‐reported emotional symptoms at age 7 years and so on. Interdependencies between subscales were not accounted for.

To meet the second objective, we examined the proportion of children with SWRD with a diagnosis ASD and/or ADHD. A chi‐squared test of association was used to determine whether the proportion of children with these diagnoses differed between the SWRD group and the rest of the population. As a sensitivity analysis, all analyses mentioned previously were then repeated as conducted on children with and without LWRA.

Finally, we assessed whether children in the group with SWRD had recognized SEN as reported by their teachers at age 7 years, and if so how their needs had been classified. The behavioural categories into which children with SWRD (as identified by the current study) were categorized by teachers were plotted as bar graphs for comparison. A linear regression *t*‐test was used to compare the level of co‐occurring behavioural problems in children with SWRD both with and without recognized SEN. We also compared word reading scores to see if severity of reading difficulty predicted recognition of SEN.

## Results

The Millennium Cohort sample analysed comprised of 1 2631 children at sweep 4 (children with both word reading and GCA scores). Of these children, 284 had SWRD (2.24%) according to our definition. The mean score for GCA was 104.9, SD = 11.4. The SWRD group differed little in measured GCA to the non‐SWRD group (103.6; SD = 15.04, compared with 105.6; SD = 11.33). Nevertheless, there was a large difference in their word reading scores (SWRD 70.8; SD = 11.47, compared with Non‐SWRD 112.3; SD = 16.66). All the SWRD individuals had measured GCA > 88; we therefore regard them as being unexpected underachievers. The SWRD group was 77.9% male, giving a boy : girl ratio of 3.6:1.

Table 2 shows descriptive statistics and comparison of mean scores for SDQ measures in children with and without specific word reading difficulties at age 11 years. The mean scores of all the measures of behaviour were significantly worse in the group of children with SWRD than they were in those without at the 1% level according to the reports of both teachers and parents. This was also true to initial behavioural difficulties at age 7 years: all SDQ subscales at age 7 years showed significantly more impairment for those with SWRD compared with those without (all at *p* < 0.001).

**Table 2:** Descriptive statistics for Strengths and Difficulties Questionnaire (SDQ) measures at age 7 years and age 11 years in children with and without specific word reading difficulties (SWRD) from the Millennium Cohort Study, and comparison of levels of behavioural problems between groups

	Age 11 years				Age 7 years				Age 11 years adjusted for initial SDQ behavioural difficulty at age 7		
SDQ Measure^a^	SWRD	No SWRD	*t*	*p*	SWRD	No SWRD	*t*	*p*	Coefficient (95% CI)	*t*	*p*
Parent report	Mean (standard error [S])		Unadjusted		Mean (SE)		Unadjusted				
*n*	206	9843	10049		281	12188	12469		9922		
Emotional symptoms	3.09 (0.38)	1.85 (0.03)	3.28	<0.001	2.45 (0.18)	1.46 (0.02)	5.34	<0.001	0.61 (0.10, 1.13)	2.36	0.019
Conduct problems	2.92 (0.33)	1.39 (0.02)	4.61	<0.001	2.44 (0.17)	1.34 (0.02)	6.67	<0.001	0.78 (0.41, 1.15)	4.14	<0.001
Hyperactivity	5.42 (0.37)	3.07 (0.04)	6.27	<0.001	5.00 (0.23)	3.23 (0.03)	7.48	<0.001	1.06 (0.55, 1.57)	4.06	<0.001
Peer problems	2.57 (0.20)	1.33 (0.02)	6.18	<0.001	2.01 (0.15)	1.15 (0.02)	5.51	<0.001	1.24 (0.56, 1.93)	3.59	<0.001
Prosocial behaviour	7.78 (0.29)	8.79 (0.02)	−3.42	0.001	7.64 (0.15)	8.41 (0.03)	−5.14	<0.001	−0.73 (−1.20, −0.30)	−3.00	0.003
Impact					1.27 (0.21)	0.23 (0.01)	4.83	<0.001			
Teacher report	Mean (SE)		Unadjusted		Mean (SE)		Unadjusted		Adjusted for initial SDQ behaviour at age 7 years		
*n*	94	6157	6251		174	7837	8011		4143		
Emotional symptoms	2.82 (0.28)	1.37 (0.03)	5.15	<0.001	2.09 (0.21)	1.43 (0.02)	3.16	0.002	1.50 (0.80, 2.20)	4.22	<0.001
Conduct problems	2.52 (0.45)	0.68 (0.02)	4.12	<0.001	2.02 (0.21)	0.76 (0.02)	6.09	<0.001	0.80 (0.20, 1.41)	2.61	0.009
Hyperactivity	5.40 (0.56)	2.25 (0.04)	5.61	<0.001	5.30 (0.30)	2.81 (0.04)	8.37	<0.001	1.25 (0.48, 2.03)	3.19	0.002
Peer problems	2.57 (0.36)	1.15 (0.03)	3.87	<0.001	2.04 (0.18)	1.11 (0.02)	5.04	<0.001	0.54 (‐0.16, 1.24)	1.53	0.13
Prosocial behaviour	6.93 (0.27)	8.10 (0.04)	−4.24	<0.001	6.23 (0.23)	7.83 (0.04)	−6.92	<0.001	−0.18 (−0.84, 0.47)	−0.55	0.58
Impact					1.41 (0.17)	0.33 (0.01)	6.42	<0.001			

a Increasing score = increasing impairment except for prosocial scores where the scale measures strength.

On adjusting for initial behavioural difficulties at age 7 years, specific reading difficulties no longer predicted teacher‐reported peer problems or poor prosocial behaviour at age 11 years (Table 2). SWRD was, however, associated with elevated behavioural difficulties at age 11 years in all domains according to parent report, and with elevated levels of emotional problems, hyperactivity and conduct issues according to teachers, even having controlled for greater levels of baseline difficulties in this group. Thus the increase in behavioural difficulties between ages 7 and 11 years was greater for the group with SWRD than for typical readers.

#### LWRA‐ sensitivity analysis

There were 344 children with LWRA (2.57%) according to our non‐discrepant definition. One hundred and seventy‐nine children were in both SWRD and LWRA categories, 63% of the SWRD group. For age adjusted word reading, the mean score for children without LWRA was 112.5, SD = 16.8, and the mean score for those with LWRA was 66.4, SD = 6.11. The LWRA group was 72.4% male, giving a boy : girl ratio of 2.7:1.

At sweep 4, age 7 years, all SDQ subscales tested, including impact, showed significantly more impairment for those with LWRA compared with those without (all at *p* < 0.001). At 11 years of age, the same was true: the means scores of all the measures of behaviour were significantly worse in the group of children with LWRA than those without at the 1% level according to the reports of both teachers and parents. After adjusting for initial behaviour at age 7 years, the results for children with LWRA at 11 years were strikingly similar to results for the SWRD category. All SDQ behavioural difficulties had significantly increased (at the 1% level) for children with low word reading attainment compared with those without. The only exception to this was teacher‐reported prosocial behaviour that showed no difference between the two groups; –*t* = −0.45 (*n* = 4313), *p* = 0.650. Teacher‐reported peer problems were significantly worse at the 5%, but not at the 1% level; *t* = 2.40 (*n* = 4314), *p* = 0.017.

To summarize, both parents and teachers reported elevated levels of behavioural problems for children with SWRD and LWRA in all domains. On adjusting for initial behavioural difficulties at age 7 years, specific word reading difficulties no longer predicted teacher‐reported peer problems or poor prosocial behaviour at age 11 years (Table 2). SWRD was, however, associated with elevated behavioural difficulties of all types examined, according to parent report, and with elevated levels of emotional problems, hyperactivity and conduct issues according to teachers, even having controlled for greater levels of baseline. All of these behavioural problems were also significantly elevated in the LWRA group, with the addition of significantly higher teacher‐reported peer problems for children with LWRA.

#### Autism spectrum disorder and attention deficit hyperactivity disorder diagnosis

In the MCS population as a whole, 1.7% of children had a diagnosis of ASD and 1.5% a diagnosis of ADHD according to parent report. For children with specific word reading difficulties, 4.6% had a parent‐reported diagnosis of ASD (*n* = 13), and for LWRA, slightly more, 5.6% had ASD. In addition, 5.3% with SWRD had a parent‐reported diagnosis of ADHD (*n* = 15) and 21% of those with LWRA. The proportion of children with ADHD and ASD that had LWRD was 13% and 12%, respectively. The number of children with both ASD and ADHD was significantly higher in the sample with word reading difficulties than for those with typical reading ability (for ADHD, Pearson *χ* ^2^(1) = 42.79, *p* < 0.001; for ASD, Pearson *χ* ^2^(1) = 35.02, *p* < 0.001).

**Figure 2:**
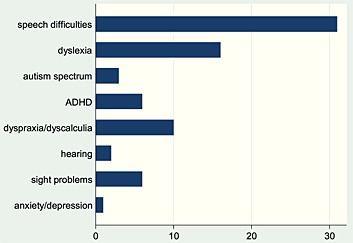
Number of children with specific word reading difficulties in each area of need (only given for children with special education needs) as classified by teachers when children were age 7 years.

Finally, we assessed whether children had recognized SEN as reported by their teachers at age 7 years. For children with SWRD and recognized SEN, we plotted the number in each ‘category of need’ as reported by teachers (Figure 2). Seventy per cent of children with SWRD had recognized SEN at age 7 years; amongst typical readers, 21% had recognized SEN. Speech and language difficulties were identified in a large minority of the group with specific word reading difficulties (36%). ‘Dyslexia’ was recognized in approximately 10% of children with SWRD. Table 3 shows descriptive statistics and comparison of mean scores for SDQ measures in children with SWRD with and without recognized SEN at age 7 years. Thirty per cent of children with SWRD did not have recognized SEN (*n* = 54). All behavioural SDQ measures except emotional symptoms as rated by teachers were significantly elevated in the group with recognized SEN. In addition, this group had more severe reading difficulties (Table 3).

**Table 3:** Descriptive statistics and comparison of mean scores for Strengths and Difficulties Questionnaire (SDQ) measures in children with specific word reading difficulties with and without recognized Special Educational Need at age 7 years

	SEN (*n* = 118) mean	No SEN (*n* = 54) mean	*n*	*t*	*p*
SDQ Measures					
Parent report					
Prosocial behaviour	7.53	8.56	170	−2.64	0.009
Peer problems	2.32	1.18	170	3.63	<0.001
Hyperactivity	5.55	2.97	170	4.65	<0.001
Conduct problems	2.56	1.17	170	3.83	<0.001
Emotional symptoms	2.81	1.71	170	4.54	<0.001
Impact	1.78	0.23	170	4.54	<0.001
Teacher report					
Prosocial behaviour	5.71	7.78	121	−4.65	<0.001
Peer problems	2.30	1.38	121	2.35	0.020
Hyperactivity	5.96	3.35	121	4.12	<0.001
Conduct problems	2.26	1.31	121	2.02	0.046
Emotional symptoms	2.24	1.72	121	1.19	0.235
Impact	0.52	1.74	121	3.75	<0.001
Raw word reading score	69.85	77.40	170	−2.64	0.009

Increasing score = increasing impairment except for prosocial scores where the scale measures strength–*t*‐test for recognized Special Educational Need (SEN) versus no recognition.

## Discussion

Our results suggest specific word reading difficulties fall at the extreme end of a roughly normal distribution of a discrepancy trait within the population (Figure 1). In other words, our findings confirm more recent research showing no sharp delineation between SWRD and non‐SWRD readers (Shaywitz 1998). This was in contrast to Rutter and Yule (1975) who found ‘a hump’ in the group with unexpectedly poor word reading leading them to suggest there could be a bimodal distribution.

The SWRD group has 77.9% men compared with 76.7% men in the earlier Isle of Wight study. Thus, SWRD gender boy : girl ratio was 3.6:1, in line with the findings from Rutter and Yule (1975) of a 3.3:1 boy : girl ratio.

Our unadjusted analysis suggests that there are elevated levels of behavioural difficulties in children with specific word reading difficulties. These findings encompassed a broad spectrum of behaviour captured by the SDQ. Clearly, children with SWRD had more difficulties with peer relationships, more emotional and conduct problems, displayed less prosocial behaviour and were rated as more hyperactive and inattentive. Our findings showing co‐occurrence of behavioural and specific word reading difficulties correspond not only with older UK studies such as the Isle of Wight cohort, but also with recent US studies (Morgan *et al.,* 2008; Dahle, Knivsberg, & Andreassen, 2011). In particular, our study concurs with the findings of Dahle *et al.* (2011) where parents reported more social problems in dyslexic children than did teachers. At age 7 years, the impact of behavioural difficulties on parents, teachers and children themselves in the MCS sample was also greater for children with SWRD. This and other studies have shown reading problems have a troubling impact in areas other than academic functioning (Riddick, 2000; McGee *et al.,* 1986; Morgan *et al.,* 2008).

The fact that elevated levels of behavioural problems occurred according to both teacher and parent reports (at home and at school) and in both discrepant and non‐discrepant groups suggests elevated problem behaviour is widespread and pervasive across settings. This kind of information is important for recognizing that SWRD may co‐occur with other problem behaviour, and has implications for planning and implementing counteractive programmes. Assessment that recognizes each child's specific functional difficulties in both the academic and behavioural spheres is therefore likely to be the most effective way of assessing a child's social and educational needs (Hollenweger, 2012; Norwich, 2014).

Findings show both ASD and ADHD were diagnosed significantly more often in the SWRD/LWRA difficulties groups. These findings are consistent with a plethora of studies that have demonstrated that reading difficulties and ADHD and its symptoms of hyperactivity /inattention frequently co‐occur (Bental & Tirosh, 2007; Frith, 1998; Gooch, Snowling, & Hulme, 2011; Nydén, Gillberg, Hjelmquist, & Heiman, 1999; Pennington, 2006; Paloyelis *et al.,* 2010) and a smaller group of studies that found co‐occurring ASD and reading difficulty (e.g*.* Nydén *et al.,* 1999). Higher rates of diagnosis for children with LWRA were probably due to associated intellectual disability. Alternatively, it is possible that the children who received low GCA scores at age 5 years had problems in the assessment per se (due to behavioural problems/executive control problems) and not (only) intellectual ability. The proportion of children with ADHD who had co‐occurring reading difficulties was lower than expected according to other estimates (see Cheung *et al.,* 2012). This may have been because we used a stringent cutoff for reading difficulties, and utilized parent‐report of ADHD diagnosis at 7 years old when many children are yet to be identified.

There have been several theories as to why developmental delays and specific word reading and other disorders co‐occur. First, genetic pleiotropic effects have been implicated. One genetic anomaly may lead to atypical neurological development, in turn manifesting as multiple behavioural difficulties (Reiersen *et al.,* 2008). Similarly, genetic predisposition combined with an early environmental insult or common environmental exposure may affect many developmental outcomes (Finlay & Miller, 1993; Porterfield, 1994; Richardson, 2006). There have also been models at later stages of childhood where one psychological impairment serves as a ‘gateway’ spawning another difficulty (Frith & Happé, 1998). So for example, if a child has a communication deficit, this might lead to social difficulties, or inattention/hyperactivity may lead to reading difficulties. Other theorists have suggested one underlying psychological deficit, such as slow naming/processing speed, may underlie a range of behavioural and cognitive difficulties, including reading difficulties commonly described as dyslexia (Bental & Tirosh, 2007).

Our findings illustrate the possibility that reading difficulties are primary, and behavioural problems might flow from frustration and inability to deal with task demands. Focused work is harder for children with specific reading difficulty resulting in them having to struggle to attend in the classroom. More research to examine the longitudinal trajectories of behavioural difficulties and their relationship with specific and non‐specific word reading is recommended. It must be acknowledged, too, that genetic effects can be seen at any time in the life course, as they interact with environment. Reading ability has a complex multifactorial genetic predisposition, which is potentially influenced by later environmental factors.

All behavioural SDQ measures, except emotional symptoms as rated by teachers, were significantly elevated in the group with recognized SEN, as opposed to without recognition. This suggests children with a higher level of behavioural problems are more likely to be recognized. The extent of reading difficulties also predicted recognition, as might be expected. Further research tracking the outcomes for the group with identified SEN as opposed to a group matched on impairment without identified SEN should highlight effectiveness of school provision.

It is somewhat reassuring that 70% of children with specific word reading difficulties had recognized SEN and the categories into which these were classed were varied, covering a wide range of behavioural difficulties. Speech and language difficulties remained the mode category. Numerous studies suggest speech and language/communication impairment is a major risk factor for reading difficulty, and subsequently the ability to link spoken language and letters may be synonymous with the development of word reading skills (Snowling, Gallagher, & Frith, 2003).

The fact that a minority of children with SWRD were recognized by teachers as having ‘dyslexia’ may be due to unwillingness to use the label at such a young age. Dyslexia is characterized as difficulties learning to read and spell. But it may be that the behaviour that challenges and burdens teachers may be exacerbated from a failure to recognize specific word reading disability. If teachers were to see co‐occurring behavioural difficulties as a warning of potential reading disability this could lead to earlier and more focussed intervention at school and less culpability for slow reading/spelling achievement and accompanying behavioural problems.

Assessment and recognition of functional problems, not just academic limitation would promote a more holistic portrait of a child both in and out of school. By functional difficulties, we mean the practical abilities to cope with day‐to‐day life and real world situations. Although our study considers difficulty in the emotional or behavioural domain, functional difficulties have previously included to any difficulty in emotional, behavioural sensory, movement and cognitive domains (Pastor, Reuben, & Loeb, 2009). The International Classification of Functioning, Disability and Health, (World Health Organization (Ed.), 2007) sets out level of functioning as a measure of both what a person can do in a standard environment (their level of capacity) and what they actually do in their usual environment (their level of performance).

Functioning can reduce involvement in daily activities and can lead to school absences, and have an impact beyond the classroom into the family. The categorical diagnosis of ‘dyslexia’ or ‘specific word reading difficulties’ may not be adequate to describe functional difficulties that may be experienced by children and their families with word reading problems. Furthermore, diagnosis on its own does not account for the inter‐relationship of developmental processes and the effect of environmental and social circumstances on a child's performance (Lollar, Hartzell, & Evans, 2012).

### Limitations

Our study had a number of limitations. First, the GCA measure was recorded at mean age of 5 years and the word reading score at mean age of 7 years; although *t*‐scores utilized were age adjusted, it would have been better to have used scores measured at the same age in discrepancy regression and definition of the group with specific word reading difficulty. There is some evidence to suggest that once children start school, IQ measures tend to stabilize (Asbury & Plomin, 2014). Schneider, Niklas, and Schmiedeler (2014) cite a correlation of *r* = 0.77 for ages 8 and 17 years. Our sensitivity analysis, which did not require GCA measures compensated for this, as results were very similar, we can be more confident the findings are robust.

SDQ measures were parent and teacher reported rather than measures derived from objective observations of behaviour. Therefore they could have been subject to reporter bias. Teacher‐ratings at sweeps 4 and 5 had lower response rates than ratings from parents. It is not known how these omissions may have affected the representativeness of our results, although the reasonable sample size of over 4,000 teachers and weighting to account for attrition in the parent‐sample provide an argument for confidence.

#### Conclusion

Our findings suggest SWRD difficulty influences behavioural problems in later childhood and/or vice versa. SWRD preceded behavioural difficulties, although we cannot infer causality as behavioural problems may have exacerbated SWRD. Findings point to ‘gateway’ theories as one mechanism underlying co‐occurring difficulties. An overarching complex system is likely in which genes, environment and social context interact to determine outcome. Assessment that identifies each individual child's specific functional difficulties rather than relying on diagnostic categories would seem the most effective way of providing classroom social and educational intervention.

### Practitioner Points

- Co‐occurring behavioural problems are common for children with specific word reading difficulties.
- The impact of co‐occurring behavioural difficulties is significant both at home and in the classroom.
- Findings suggest specific word reading difficulties may lead to behavioural difficulties, although causality is likely to be complex and multifactorial.
- Assessment that identifies each individual child's specific functional difficulties is recommended as the most effective way to providing support.

## Conflict of Interest

None of the authors have any conflict of interest.
